# Neuroinflammation in Recent Onset Mental Health Disorders – Developing Multi-level Signatures of Early-stage Depression and Psychosis in Young Adults

**DOI:** 10.1192/j.eurpsy.2024.147

**Published:** 2024-08-27

**Authors:** D. Popovic, C. Weyer, A. Ruef, D. Dwyer, S. L. Griffiths, P. A. Lalousis, N. Koutsouleris, R. Upthegrove

**Affiliations:** ^1^Max Planck Institute of Psychiatry; ^2^Psychiatry and Psychotherapy, Ludwig-Maximilian-University, Munich, Germany; ^3^Centre for Youth Mental Health, University of Melbourne, Melbourne, Australia; ^4^School of Psychology, University of Birmingham, Birmingham; ^5^Institute of Psychiatry, Psychology & Neuroscience, King’s College London, London, United Kingdom

## Abstract

**Introduction:**

An early and comprehensive neurobiological characterization of severe mental disorders could elucidate mechanistic pathways, aid the development of novel therapeutics, and therefore enable timely and targeted intervention in at-risk youth and young adults. Therefore, we present an unsupervised transdiagnostic machine learning approach to investigate shared and distinct patterns of early-stage depressive and psychotic disorders on multiple clinical and neurobiological levels.

**Objectives:**

To derive multi-level neurobiological and clinical signatures of early-stage affective and psychotic disorders in adolescents and young adults.

**Methods:**

From the multicenter prospective European PRONIA cohort, we acquired data from 678 individuals (51% female) comprising young, minimally medicated in- and outpatients with clinical high-risk (CHR) states for psychosis, with recent-onset depression (ROD) or psychosis (ROP), and healthy control (HC) individuals. Within repeated nested cross-validation frameworks, we employed Sparse Partial Least Squares Analysis to detect associations between blood markers and grey matter volume (GMV), followed by support vector machine prediction of these signatures using biographical, clinical, neurocognitive, proteomic, and functional data.

**Results:**

Our results demonstrated a psychosis staging signature separating ROP from CHR individuals via GMV patterns in the cortico-thalamo-cerebellar circuitry with a blood marker set of elevated of IL-6, TNF-α and CRP (ρ = 0.272; P = 0.002). A depression signature separated ROD from HC individuals via altered GMV in the limbic system with a blood marker set of elevated IL-1ß, IL-2, IL-4, S100B and BDNF (ρ = 0.186; P = 0.021). Only the psychosis staging signature showed a distinct proteomic enrichment regarding innate immune response, abnormal neutrophil function, cellular senescence, and anti-inflammatory drugs (Balanced Accuracy (BAC) = 87.73%; Area Under the Curve (AUC) = 0.94). Childhood trauma differentially predicted psychosis and depression signatures, while past level of functioning, personality and quality of life was predictive of both signatures (BAC = 67.19-78.00%; AUC = 0.71-0.83).

**Image:**

**Figure:**
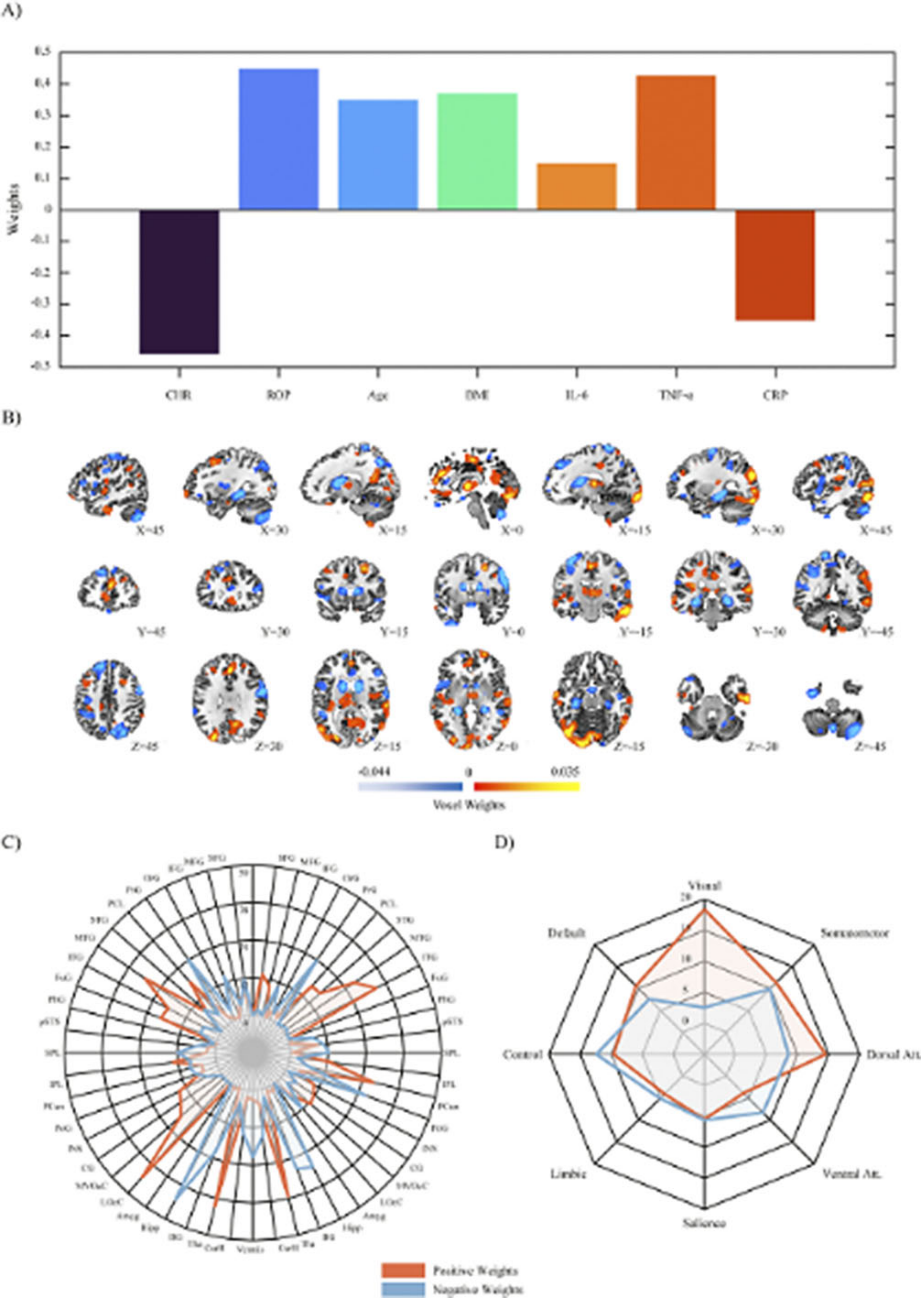

**Image 2:**

**Figure:**
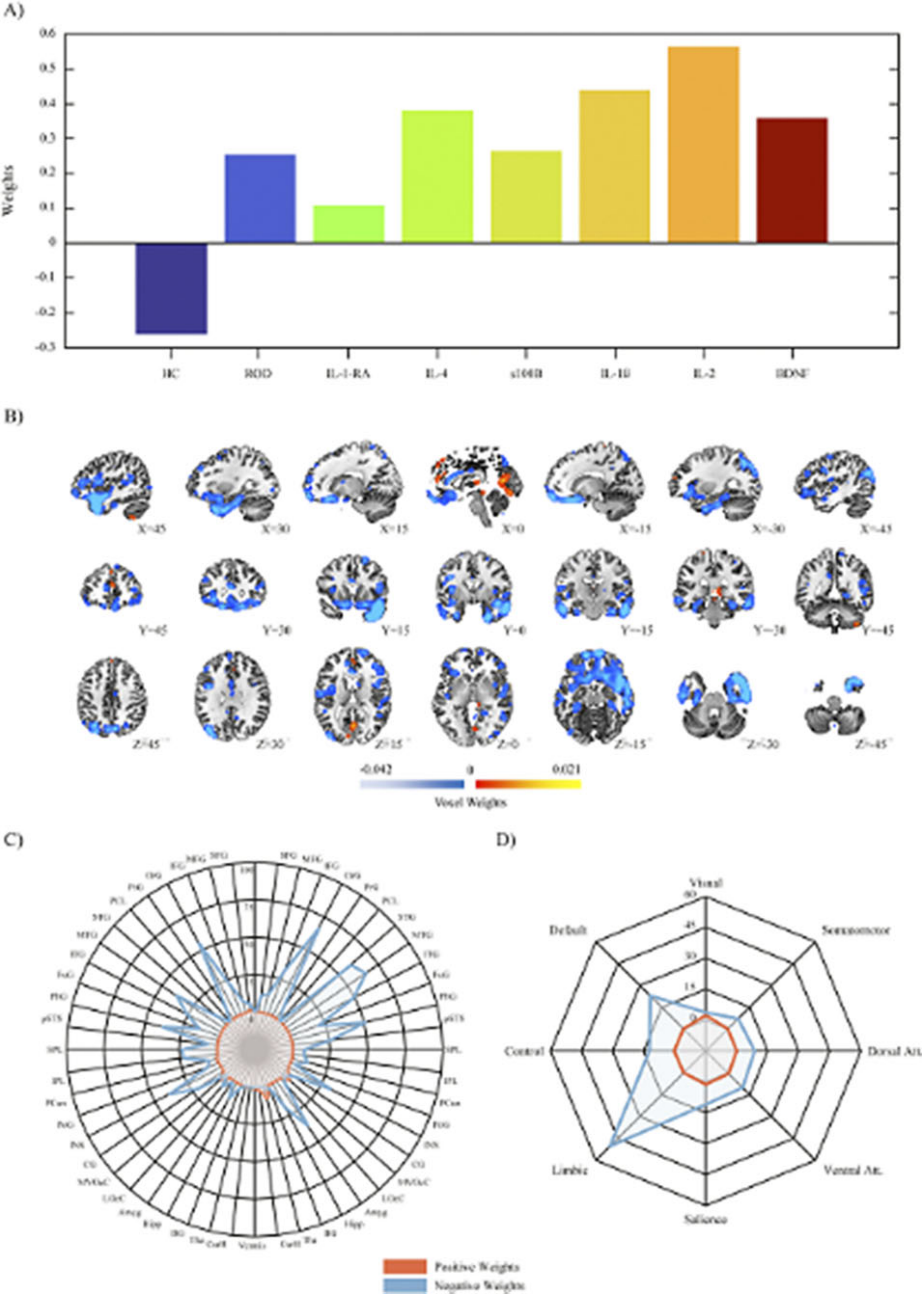

**Image 3:**

**Figure:**
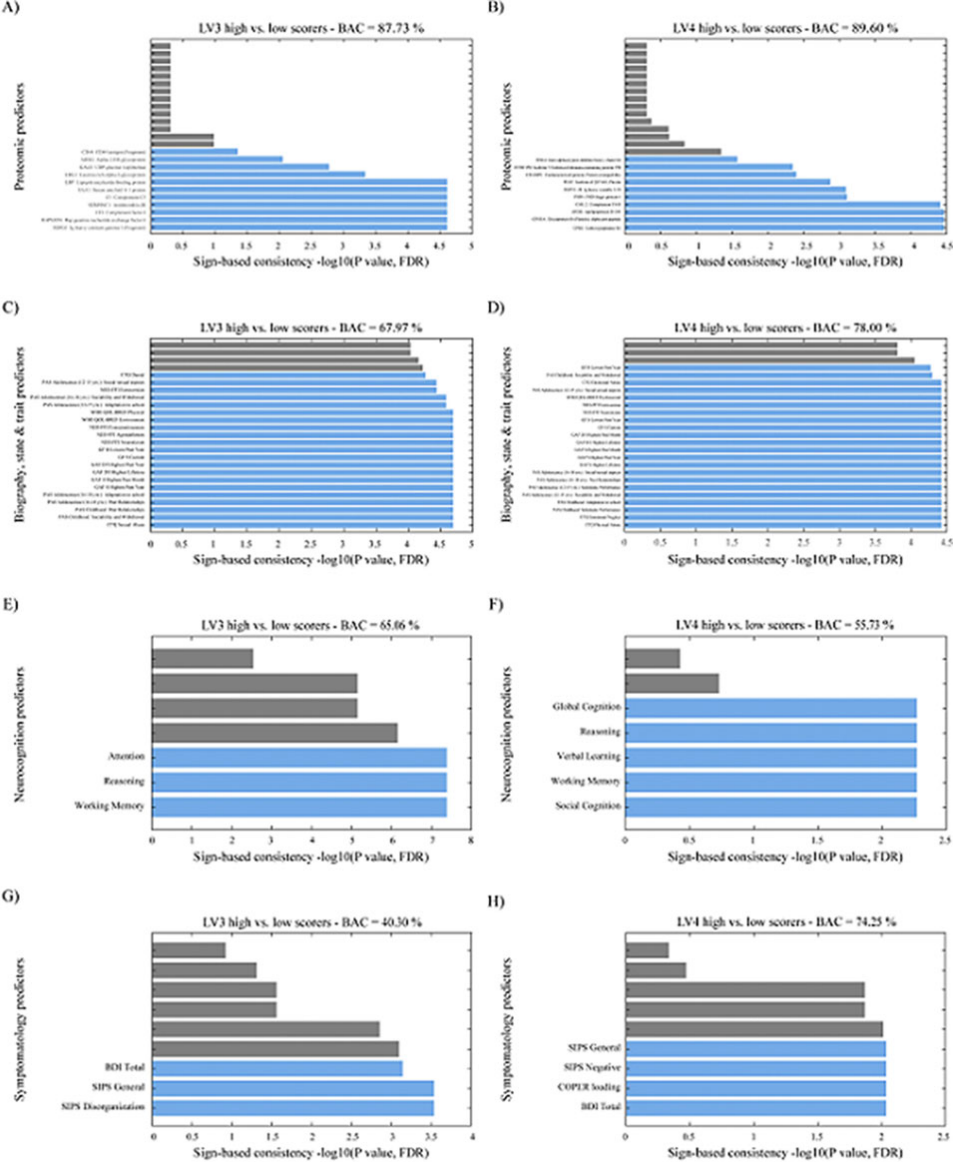

**Conclusions:**

Psychosis and depression exhibit distinct multi-level signatures evident in early disease stages. Enhanced insight into these signatures could help delineate individual trajectories and potentially new mechanisms for pharmacological treatment.

**Disclosure of Interest:**

None Declared

